# Elite Diving Athlete With Traumatic Growth Plate Injury of the Proximal Humerus: A Case Report

**DOI:** 10.7759/cureus.20293

**Published:** 2021-12-09

**Authors:** Reo Asai, Masaki Tatsumura, Yuta Tsukagoshi, Masashi Yamazaki

**Affiliations:** 1 Department of Orthopaedics, Tsukuba Medical Center Hospital, Tsukuba, JPN; 2 Department of Orthopaedic Surgery and Sports Medicine, Tsukuba University Hospital Mito Clinical Education and Training Center, Mito Kyodo General Hospital, Mito, JPN; 3 Department of Paediatric Orthopaedic Surgery, Ibaraki Children's Hospital, Mito, JPN; 4 Department of Orthopaedic Surgery, Faculty of Medicine, University of Tsukuba, Tsukuba, JPN

**Keywords:** mri, proximal humerus, growth plate injury, shoulder pain, competitive diving

## Abstract

Growth plate injury of the proximal humerus is rare. We herein report a traumatic growth plate injury of the proximal humerus in an elite diving athlete. A 16-year-old female diving athlete injured her left shoulder during 7.5 m platform hands-first diving practice. At the first visit, she presented with upper left shoulder tenderness and left shoulder range-of-motion limitation. There was no fracture or dislocation on X-ray and computed tomography (CT), but magnetic resonance imaging (MRI) showed a high-intensity zone in the left lateral epiphysis of the proximal humerus. We treated her conservatively by rest with sling and rehabilitation. She partially restarted diving practice five weeks post-injury and returned to competition eight weeks post-injury. Even if there is no sign of fracture or dislocation, we should consider MRI for patients who are before the age of growth plate closure.

## Introduction

Growth plate injury of the proximal humerus is a rare injury [1] that can cause early growth plate closure and lead to angular deformity or length discrepancy [2]. Despite the high injury incidence, injury reports in competitive diving are few [3], and we herein report a case of traumatic growth plate injury of the proximal humerus for the first time in an elite diving athlete.

## Case presentation

**Figure 1:**
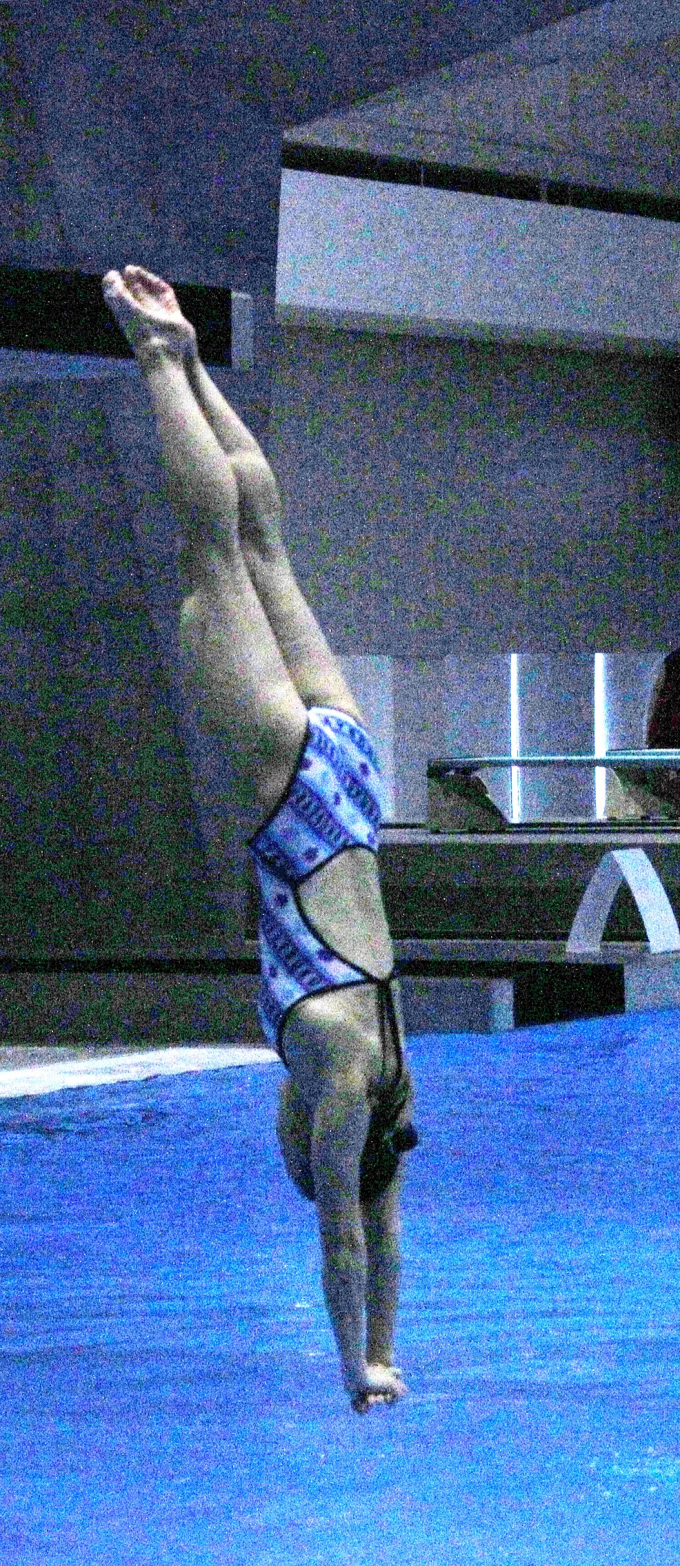
Water entry at hands-first diving.

**Figure 2:**
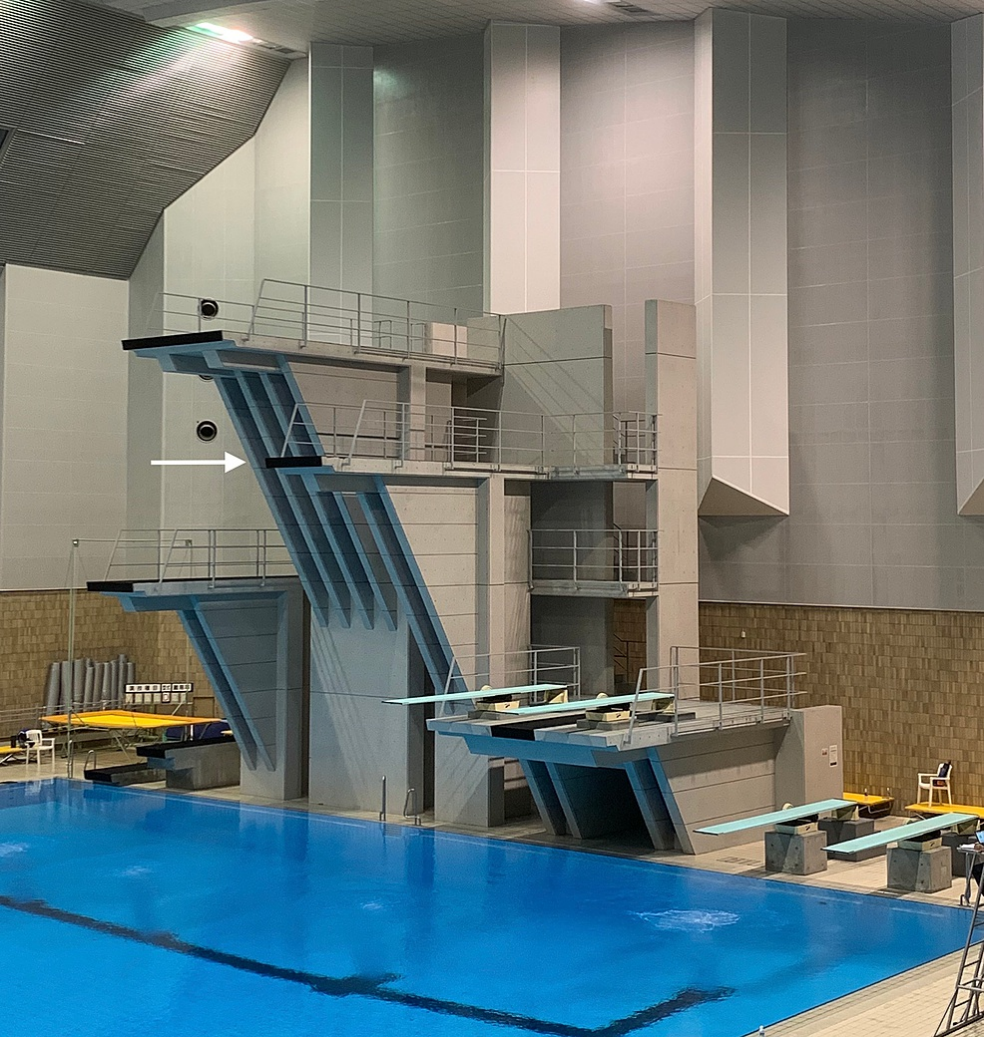
The diving pool with platforms and springboards. The platforms are set at 5 m, 7.5 m (white arrow), and 10 m. The springboards are set at 1 m and 3 m.

A 16-year-old female diving athlete, with no past dislocation or pain history, injured her left shoulder during diving practice (Figure 1). When diving from a 7.5 m platform (Figure 2), incomplete hands-first entry forced abduction and external rotation of her left shoulder joint. Left shoulder pain occurred soon after and did not improve, so she visited our hospital two days post-injury.

**Figure 3:**
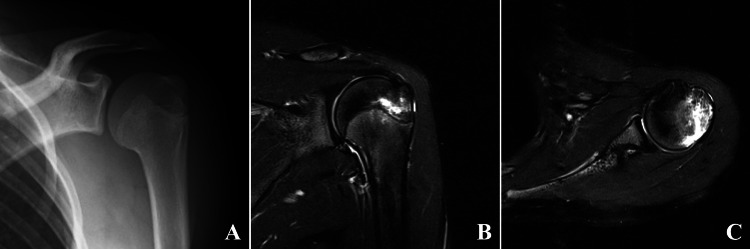
X-ray and MRI of the left shoulder at the first visit. A, Frontal view of the X-ray. B, Coronary MRI STIR slice. C, Axial MRI STIR slice. MRI, magnetic resonance imaging; STIR, short-tau inversion recovery.

At the first visit, she presented with upper left shoulder tenderness and left shoulder range of motion (ROM) limitation with passive flexion, abduction, and external rotation angles of 160°, 150°, and 80°, respectively due to pain. Apprehension tests were negative. We were not able to detect any fractures or dislocation on X-ray and computed tomography (CT) compared with the contralateral side, but magnetic resonance imaging (MRI) short-tau inversion recovery (STIR) imaging showed a high-intensity zone in the left lateral epiphysis of the proximal humerus (Figure 3). No bony Bankart lesions on CT or Hill-Sachs lesions were apparent. Because she had never had similar symptoms in the past, including on the contralateral side, we diagnosed her with traumatic growth plate injury caused by a single external force. Rest with sling and rehabilitation were initiated as conservative therapy. As a rehabilitation, muscle training and active ROM exercise within pain-free range were initiated for the first two weeks. After two weeks, the ROM ranges had gradually increased.

**Figure 4:**
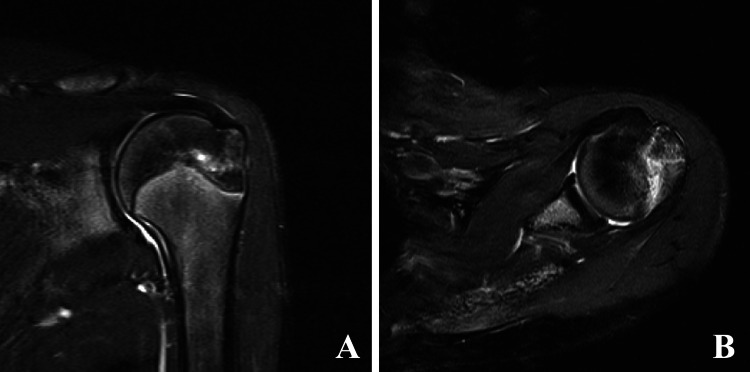
MRI of the left shoulder at six weeks post-injury. A, Coronary MRI STIR slice. B, Axial MRI STIR slice. MRI, magnetic resonance imaging; STIR, short-tau inversion recovery.

At her visit four weeks post-injury, the left shoulder tenderness disappeared. The passive flexion angle was 180°, and the high-intensity zone in MRI STIR imaging decreased. She restarted handstand and feet-first diving practice from four and five weeks post-injury, respectively. The high-intensity zone in MRI STIR imaging had almost disappeared after six weeks post-injury (Figure 4), and she resumed hands-first diving practice. Moreover, she returned to the competition in springboard diving at eight weeks post-injury.

At her visit 12 weeks post-injury, shoulder ROM limitations were completely resolved with passive flexion, abduction, and external rotation angles of 200°, 180°, and 110°, respectively; therefore, she restarted platform diving practice. Six months post-injury, she won a prize at a national diving competition. After more than two years of follow-up, there has been no reoccurrence of the symptoms.

## Discussion

The mechanism of proximal humeral injury can be categorized into two types: first, by direct force caused by falling on the shoulder; second, by the indirect force caused when falling on an outstretched hand [2]. In competitive diving, athletes enter hands-first with outstretched hands; thus, we assume that the growth plate injury occurred by the axial impact experienced during hands-first diving.

When athletes perform hands-first diving from a 10 m platform, they reach a speed of about 14.1 m/s (51 km/h) at the moment of entering the water and decelerate to 9.25 m/s (33 km/h) immediately. The decrease of speed is 4.85 m/s, and this is the same amount of impact we receive when we fall from 1.2 m to a hard surface [4]. Moreover, the shoulder is, in most cases, the endpoint to receive the water entry impact. In successful hands-first diving, the shoulder can absorb much of the water entry axial loading [5]. In failed hands-first diving, however, the shoulder may not be able to absorb the axial loading and is damaged. In this case, the athlete dived from a 7.5 m platform, but we expect that the axial impact the shoulder received was strong enough to cause the growth plate injury of the proximal humerus.

In competitive diving, injuries around the shoulder, lower back, and wrist are the most frequently reported [3,6,7]. Among shoulder-related injuries, shoulder joint subluxations and dislocations have been reported in diving athletes in the past [8]. Subluxation should be considered in this case; however, based on the negative apprehension tests and the absence of Bankart or Hill-Sachs lesions, we determined that subluxation is negative. Proximal humeral epiphysiolysis, which is known as Little League shoulder, shows similar MRI findings, and thus, should also be differentiated. Typically, it is common among young overthrowing athletes presenting with chronic shoulder pain, and physeal widening on X-ray is distinctive [9]. However, we consider it to be negative for the following reasons: first, competitive diving is not an overthrowing sport; second, no physeal widening was apparent compared to the contralateral side; third, the point of injury was clear; and fourth, the pain had not occurred before or recurred since.

Although injury reports in competitive diving are relatively few, the injury incidence is high. Between 92% and 100% of diving athletes reported injuries that required at least one week out of training [3]. In addition, the injury cases increased as the years of training and competition increased, were more common during practice than during competition, and were more frequent when diving from platforms than springboards. There was no gender difference, but water entry accounted for a large part of the injury mechanism among diving athletes [3]. In this case, a diving athlete with competition experiences from an early age was injured when entering the water during platform diving practice. Thus, this situation can be considered as having a high risk of injury.

Growth plate injury of the proximal humerus is a rare injury, representing 3% of all growth plate injuries in children [1]. Despite the low occurrence rate, growth plate injury of the proximal humerus can cause early growth plate closure, and thereby lead to angular deformity or length discrepancy [2]. Therefore, we must consider growth plate injury of the proximal humerus in the differential diagnosis.

The most common radiographic injury pattern of growth plate injury of the proximal humerus is reported to be type II injuries based on the Salter-Harris classification [2]. If the fracture line is apparent on X-ray, it can be diagnosed with this finding alone. Sometimes, however, X-ray findings are not clear; therefore, MRI, which is an excellent imaging method to depict epiphyseal injuries [10], is a better imaging option in cases like the one presented here.

## Conclusions

We must consider growth plate injury of the proximal humerus in the differential diagnosis of shoulder pain. Subluxation and proximal humeral epiphysiolysis should be differentiated based on the patient's past medical history, the point of injury, physical examination, and imaging findings. Even if there is no sign of fracture or dislocation in patients with a suspected shoulder injury, we should consider taking an MRI if the patients are before the typical age of growth plate closure.
